# Perspectives on COVID-19 Vaccines and Its Hesitancy Among Jordanian Population

**DOI:** 10.7759/cureus.26337

**Published:** 2022-06-26

**Authors:** Mohamad Abdullah, Awni D Shahait, Rami Qaisieh, Rakan Wleidat, Mohammad Al-Ramahi, Ghayda Bader, Muawia O AbuRajab, Tala A Haddad, Ahmad Y Al-Omari, Mohammad S Bani Issa, Taima Bader, Sama Bani Ahmad, Hala Hani, Haya Hani, Abed AlFattah AlNsour, Basil Abdin

**Affiliations:** 1 Surgery, Sheffield Teaching Hospitals NHS Foundation Trust, Sheffield, GBR; 2 Surgery, Wayne State University School of Medicine, Detroit, USA; 3 Anesthesia, Hashemite University of Jordan, Zarqa, JOR; 4 Ophthalmology, The Jordanian Ministry of Health, Amman, JOR; 5 Orthopedics, The Jordanian Ministry of Health, Amman, JOR; 6 Internal Medicine, School of Medicine, University of Jordan, Amman, JOR; 7 Nursing, Faculty of Nursing, University of Jordan, Amman, JOR; 8 Pharmacy, Faculty of Pharmacy, University of Jordan, Amman, JOR; 9 Internal Medicine, Jordanian Royal Medical Services, Amman, JOR; 10 Internal Medicine, Al-Makassed Hospital, Jerusalem, PSE

**Keywords:** covid-19 vaccine hesitancy, covid-19 vaccine, vaccine development, vaccine hesitancy, covid-19

## Abstract

Background

Since the spread of the COVID-19 virus, governments are putting significant resources into ending the pandemic. Vaccination arises as the best solution to get back to our everyday lives. However, we are now facing vaccine hesitancy, which is a critical problem.

Methods

This cross-sectional study was conducted between December 15, 2020, and March 1, 2021, using a validated online-based questionnaire; participants were compared using the Statistical Package for the Social Sciences (SPSS) program based on multiple factors.

Results

A total of 1607 participants throughout Jordan have responded to the questionnaire, among which 880 (54.8%) have bachelor's degrees, 236 (14.7%) have a high educational level (master and doctoral), and 491 (30.5%) have a diploma or less. Although this is a non-probable sample, it is not a representative sample as, according to United Nations Educational, Scientific and Cultural Organization (UNESCO), only 33.6% of the Jordan population have a tertiary education.

Overall, 892 (55.5%) of the studied subjects had the intention to take the vaccine, distributed as follows: 156 (66.1%) of the high educational participants wanted to take the vaccine, compared to 512 (58.2%) of those who have bachelor’s degree and 224 (45.6%) of those who have diploma or less (p < 0.001). Reading scientific articles talking about the vaccines and their effects (55.6%, p < 0.001), knowing the mechanism of action (45.2%, p = 0.007), getting proper medical advice (27.2%, p < 0.001), encouraged by the increasing number of infections and deaths (39.7%, p < 0.001), and the number of people who received the vaccine (16.1%, p < 0.001) were the most critical factors that played a role in taking the vaccine by all of the studied groups.

Male gender (OR = 2.02; 95% CI = 1.54-2.64; p < 0.001), high income of more than 1000 JDs (1400 USD) (OR = 3.23; 95% CI = 2.21-4.71; p < 0.001), having an educational level of either high education (OR = 3.39; 95% CI = 2.07-5.55; p < 0.001) or bachelor degree (OR = 1.67; 95% CI = 1.25-2.24; p = 0.001), and being encouraged by the increasing number of infections and deaths caused by COVID-19 (OR = 1.97; 95% CI = 1.46-2.66; p < 0.001) were all significantly associated with the willingness to take the vaccine.

Conclusion

As the world rushes toward vaccination to end the pandemic, efforts are needed to end this phenomenon of vaccine hesitancy by enlightening people with the precise knowledge regarding the vaccine's mechanism of action, side effects, and efficacy focusing mainly on people with lower educational levels.

## Introduction

Since the World Health Organization (WHO) declared the coronavirus disease 2019 (COVID-19) as a healthcare crisis on March 11, 2020, many strategies were adopted to face it, including lockdowns, quarantine, travel restrictions, social distancing, and online learning [1]. Anxiety and psychological impacts increased, especially when people needed to wear masks and wash their hands when in contact with the outside world [2,3]. Today, as the healthcare providers suggest different solutions to counter the pandemic, vaccination is the most effective way to protect people and increase their immunity against the disease [4].

Vaccine hesitancy, which refers to the delay in acceptance or refusal of vaccines despite the availability of its services [5], is considered an increasingly worldwide phenomenon. WHO declared it in the top 10 global health threats in 2019 [6,7]. Its specific context varies across time, place, and vaccines. It depends on many factors changing from one region to another, including political views and social media effects that allow rumors and conspiracy theories [8-10].

Jordan has had more than one million COVID-19 cases like other countries affected by this global pandemic. The government established an online platform to allow citizens to register for vaccination and assess the efficiency of the vaccine. Although few people registered for the vaccine in the first two months, the number has increased daily to reach 3.5 million, and most of them registered for the vaccine in March and April 2021 when there were 8000 new COVID-19 cases per day [11].

Previous studies showed that various factors play a role in vaccine hesitancy, including medical personnel, social media, lack of trust, and, most importantly, vaccine safety [12-15]. Based on a study done in France before vaccine emergence, 25% of people who refused to take the vaccine had concerns about its safety due to its fast development [16]. Moreover, many studies were published regarding vaccine acceptance worldwide, with which Jordan showed a shallow acceptance rate of 28.4% [17,18].

This study aims to shed light on the crucial factors affecting people's attitudes toward taking the vaccine. The primary objective was to assess people's awareness and knowledge of the importance of getting vaccinated against COVID-19. The secondary goal was to find the main factors that affect and encourage people's decision to vaccinate, focusing on the sources they used to get the correct, reliable information regarding vaccination.

## Materials and methods

Study design

This cross-sectional study was conducted using an online-based questionnaire between December 15, 2020, and March 1, 2021. A total of 1607 participants aged 18 years and above who are eligible to be vaccinated were approved to participate in the study.

We used a cross-sectional design as it does not involve manipulating variables. Instead, it allows researchers to look at numerous characteristics at once (age, income, and gender) and the prevailing characteristics in each population. It can provide information about what is happening in a current population. The survey was designed to allow the participants to fill out only once to reduce duplication. 

Questionnaire

The questionnaire was designed using Google Forms, an online survey creator tool developed by Google. It was divided into four sections as follows: an introductory page (the first section) presented the aim and significance of the study and outlined the research objectives, with consent as a part of this section.

The second section outlined the demographic features of the studied population, asking about general demographic data including age, gender, income, residency, educational level, chronic illnesses, and previous history of COVID-19 among the participants and their relatives.

The third section focused on the participants' general knowledge and concerns regarding COVID-19 vaccines, their willingness to take the vaccine, and which vaccine they prefer and trust the most.

Finally, the last section investigated the factors that would affect the decision to take the vaccine, mainly by outlining the most encouraging factors for vaccination, such as reading scientific articles, knowing the vaccine's mechanism of action, and medical advice. 

Ethical approval

The study was approved by the Institutional Review Board (IRB) of the Hashemite University, Zarqa, Jordan. The IRB approval number is 6/7/2020/2021 and is titled “Comparison of post-COVID-19 vaccination complications and immunological responses of Pfizer-bitonic and Sinopharm vaccines.” The assent of participants to advance to the questionnaire was sought through informed consent after the online questionnaire's introductory page. The data was obtained anonymously for statistical purposes only without asking about personal information. 

Statistical analysis

The data were analyzed using the Statistical Package for the Social Science (SPSS) version 25.0 (IBM Corp., Armonk, NY) program. Descriptive statistics were presented as numbers (percentage) for categorical variables and mean standard deviation (SD) for numeric variables. We compare the categorical variables between the population based on their educational level divided into high education, bachelor's, and diploma or less using the Chi-square test, depending on a two-sided p-value < 0.05 for statistical significance.

The significant variables in univariate analysis were included in multiple binominal logistic regression to assess the association of each variable with the willingness to take the COVID-19 vaccine, demonstrated by the odds ratio (OR) along with the 95% confidence interval (95% CI) and a level of <0.05 as the significance threshold.

## Results

A total of 1607 participants were enrolled in this study with a mean age of 36.2 ± 12.5 years. Of the studied population, 961 (59.8%) were males. Based on the educational level, 236 (14.7%) were at a high academic level (master’s and doctoral), 880 (54.8%) have bachelor’s degrees, and 491 (30.5%) have a diploma or less degrees. Although this is a non-probable sample, it is not a representative sample as, according to the United Nations Educational, Scientific and Cultural Organization (UNESCO), only 33.6% of the Jordan population have a tertiary education. Regarding COVID-19 previous history, 354 (22%) of the participants were infected with the COVID-19 virus at least once. The demographic data and prior history of COVID-19 are shown in Table 1.

**Table 1:** Demographic features of the studied population

Characteristics		Educational level			Total	P-value
		High educational level (n = 236)	Bachelor’s degree (n = 880)	Diploma or less (n = 491)		
Age (years)					36.2 ± 12.5	0.072
Gender	Male	165 (69.9)	511 (58.1)	285 (58)	961 (59.8)	0.002
	Female	71 (30.1)	369 (41.9)	206 (42)	646 (40.2)	
Residency	North territory	25 (10.6)	101 (11.5)	64 (13)	190 (11.8)	0.003
	Middle territory	203(86)	739 (84)	382 (77.8)	1324 (82.4)	
	South territory	8 (3.4)	40 (4.5)	45 (9.2)	93 (5.8)	
Income (Jordanian Dinars)	Less than 500 JDs	39 (16.5)	404 (45.9)	339 (69)	782 (48.7)	<0.001
	500–1000 JDs	84 (35.6)	271 (30.8)	113 (23)	468 (29.1)	
	More than 1000 JDs	113 (47.9)	205 (23.3)	39 (8)	357 (22.2)	
Marital status	Married	157(66.5)	456 (51.8)	299 (60.9)	912 (56.8)	<0.001
	Single	72 (30.5)	412 (46.8)	167 (34)	651 (40.5)	
	Other	7 (3)	12 (1.4)	25 (5.1)	44 (2.7)	
Smoker		95 (40.3)	345 (39.2)	208 (42.4)	648 (40.3)	0.52
Having any chronic diseases		53 (22.5)	147 (16.7)	123 (25.1)	323 (20.1)	0.001
Having a history of COVID-19 infection		51 (21.6)	201 (22.8)	102 (20.8)	354 (22)	0.665
Having a member of family/friends infected with COVID-19		207 (87.7)	772 (87.7)	402 (81.9)	1381 (85.9)	0.010

Significant differences regarding COVID-19 vaccines were found among different educational levels (Table 2); 190 (80.5%) participants with high educational levels knew more about the vaccine compared to the 622 (70.7%) participants with bachelor's degrees and 270 (55%) participants with diploma or less degrees (p < 0.001). Similarly, the high educational level group (n = 178; 75.4%; p < 0.001) knew more about the vaccine's side effects than the bachelor’s (n = 574; 65.2%) and diploma or less degree groups (n = 261; 53.2%). Participants who have higher educational level (n = 136; 57.6%; p = 0.008) had a strong belief in the vaccine's potency to decrease the symptoms caused by the disease compared to those with bachelor’s degree (n = 430; 48.9%) and diploma or less (n = 216; 44%).

**Table 2:** General beliefs and concerns regarding COVID-19 vaccines

Characteristics	Educational level			Total		P-value
	High educational level (n = 236)	Bachelor’s degree (n = 880)	Diploma or less (n = 491)			
Knowing what COVID-19 vaccines are	190 (80.5)	622 (70.7)	270 (55)	1082 (67.3)		<0.001
Knowing the side effects of COVID-19 vaccines	178 (75.4)	574 (65.2)	261 (53.2)	1013 (63)		<0.001
Believed that COVID-19 vaccines are safe and useful	208 (88.1)	736 (83.6)	356 (72.5)	1300 (80.9)		<0.001
Believed that the COVID-19 vaccine will protect you	144 (61)	485 (55.1)	209 (42.6)	838 (52.1)		<0.001
Believed that COVID-19 vaccines will decrease the symptoms once infected	136 (57.6)	430 (48.9)	216 (44)	782 (48.7)		0.008
Believed that elderly and immunocompromised patients should take the vaccine first	178 (75.4)	663 (75.3)	314 (64)	1155 (71.9)		<0.001
Believed that COVID-19 vaccines will decrease the mortality rate worldwide	160 (67.8)	544 (61.8)	234 (47.7)	938 (58.4)		<0.001
Concerns regarding COVID-19 future after vaccination						
Willing to take the vaccine	156 (66.1)	512 (58.2)	224 (45.6)	892 (55.5)		<0.001
Feeling positive about the vaccine's success	139 (58.9)	412 (46.8)	176 (35.8)	727 (45.2)		<0.001
Will pay to take the vaccine if it is not for free	151 (64)	469 (53.3)	204 (41.5)	824 (51.3)		<0.001
Feeling positive that life will get back to normal	148 (62.7)	506 (57.5)	236 (48.1)	890 (55.4)		<0.001
The trusted vaccine to be received if willing to take						
Pfizer-BioNTech COVID-19 vaccine	126 (53.4)	46 (52.4)	210 (42.8)	797 (49.6)		0.001
Moderna COVID-19 vaccine	45 (19.1)	156 (17.7)	50 (10.2)	251 (15.6)		<0.001
Sinopharm COVID-19 vaccine	66 (28)	208 (23.6)	79 (16.1)	353 (22)		<0.001
Sputnik COVID-19 vaccine	24 (10.2)	84 (9.5)	35 (7.1)	143 (8.9)		0.232
Oxford/AstraZeneca COVID-19 vaccine	20 (8.5)	75 (8.5)	44 (9)	139 (8.6)		0.957
I do not know	66 (28)	288 (32.7)	201 (40.9)	555 (34.5)		0.001

The belief that COVID-19 vaccines are helpful and safe showed a significant result; 208 (88.1%) participants with high education believed in the vaccine's safety in comparison to 736 (83.6%) participants with bachelor's degrees and 356 (72.5%) participants with diplomas or less (p < 0.001). Other beliefs kept in the same context (p < 0.001), of the participants with high education, 144 (61%) participants believed in the strength of vaccine protection against COVID-19 virus, 178 (75.4%) thought that the elderly and immunocompromised individuals are the priority to take the vaccine, while 160 (67.8%) believed in the potency of the vaccine in decreasing deaths caused by the disease. 

Overall, 892 (55.5%) of the studied subjects had the intention to take the vaccine, distributed as follows: 156 (66.1%) of the high educational participants wanted to take the vaccine compared to 512 (58.2%) of those who have bachelor’s and 224 (45.6%) of those who have diplomas or less (p < 0.001). Of the high education group, 139 (58.9%) and 148 (62.7%) had positive feelings toward the vaccine success and returning to everyday life, respectively, compared to 412 (46.8%) and 506 (57.5%) of the bachelor's and 176 (35.8%) and 236 (48.1%) of the diploma or less groups (p < 0.001).

Interestingly, 824 (51.3%) of the studied population would pay for the vaccine once it is not free anymore (p < 0.001), distributed among the groups as follows: 151 (64%) participants with high education would pay for the vaccine compared to 469 (53.3%) of the participants with bachelor’s degree and 204 (41.5%) with a diploma or less degree. Regarding the trusted vaccine to take, if the participants had the right to choose, both high education (n = 126; 53.4%) and diploma or less (n = 210; 42.8%) groups would prefer to take the Pfizer-BioNTech vaccine (p = 0.001), whereas the bachelor’s degree group mainly did not know which vaccine to take (n = 288; 32.7%; p = 0.001).

Many variables showed significant results from the distribution of the studied population planning to take the vaccine based on their educational level (Table 3). The mean age of the participants was 37.1 ± 12.9 (p = 0.008), of which 580 (65%) were males. The lower the income of the participants, the more willing they were to take the COVID-19 vaccine. As shown in Table 3, 361 (40.5%) participants who had an income of 500 JDs (700 USD) or low would take the vaccine more, compared to 272 (30.5%) of those who had an income of 500-1000 JDs (700-1400 USD) and 259 (29%) of participants with an income of more than 1000 JDs (1400 USD) (p < 0.001). Of 156 participants with a high education willing to take the vaccine, 128 (82.1%) had not been infected before, compared to 412 (80.5%) of the bachelor’s and 181 (80.8%) of the diploma or less groups (p = 0.003). Moreover, 141 (90.4%) participants of the high education, 457 (89.3%) of the bachelor’s, and 188 (83.9%) of the diploma or less groups were willing to take the vaccine due to having a close person who got infected with COVID-19 (p = 0.009). Regarding the most encouraging factors to getting vaccinated, 496 (55.6%) of the studied subjects would take the vaccine if they had the scientific evidence about the vaccines and their effects (p < 0.001). Other factors are illustrated in Table 3.

**Table 3:** Effect of different factors on the willingness to get the COVID-19 vaccine

Characteristics		Willing to take the vaccine based on educational level (N = 892)				
		Educational level			Total	P-value
		High educational level (n = 156)	Bachelor’s degree (n = 512)	Diploma or less (n = 224)		
Age		37.6 ± 13	36.7 ± 12.8	37.8 ± 13.1	37.1 ± 12.9	0.008
Gender	Male	110 (70.5)	318 (62.1)	152 (67.9)	580 (65)	<0.001
	Female	46 (29.5)	194 (37.9)	72 (32.1)	312 (35)	
Income	Less than 500 JDs	21 (13.5)	191 (37.3)	149 (66.5)	361 (40.5)	<0.001
	500–1000 JDs	45 (28.8)	172 (33.6)	55 (24.6)	272 (30.5)	
	More than 1000 JDs	90 (57.7)	149 (29.1)	20 (8.9)	259 (29)	
Residency	North territory	8 (5.1)	53 (10.4)	33 (14.7)	94 (10.5)	<0.001
	Middle territory	144 (92.3)	444 (86.7)	178 (79.5)	766 (85.9)	
	South territory	4 (2.6)	15 (2.9)	13 (5.8)	32 (3.6)	
Having a history of chronic disease		38 (24.4)	97 (18.9)	63 (28.1)	198 (22.2)	0.062
Did not get COVID-19 infection before		128 (82.1)	412 (80.5)	181 (80.8)	721 (80.8)	0.003
Having a member of family/friends infected with COVID-19		141 (90.4)	457 (89.3)	188 (83.9)	786 (88.1)	0.009
Encouraging factors to get vaccinated						
Reading scientific articles		109 (69.9)	303 (59.2)	84 (37.5)	496 (55.6)	<0.001
Knowing the mechanism of action of the COVID-19 vaccine		74 (47.4)	248 (48.4)	81 (36.2)	403 (45.2)	0.007
Medical advice		34 (21.8)	148 (28.9)	61 (27.2)	243 (27.2)	<0.001
Social media and family effect		10 (6.4)	59 (11.5)	34 (15.2)	103 (11.5)	0.293
Past medical history		20 (12.8)	82 (16)	19 (8.5)	121 (13.6)	0.553
The high number of infections and deaths		58 (37.2)	216 (42.2)	80 (35.7)	354 (39.7)	<0.001
Duration of immunity		22 (14.1)	128 (25)	37 (16.5)	187 (21)	0.199
Knowing the side effects of the vaccine		18 (11.5)	60 (11.7)	17 (7.6)	95 (10.7)	<0.001
Increasing the number of people receiving the vaccine		21 (13.5)	90 (17.6)	33 (14.7)	144 (16.1)	0.001

Finally, we applied multivariant regression analysis to identify the impact of each variable on the willingness to take the vaccine. As shown in Table 4, male gender (OR = 2.02; 95% CI = 1.54-2.64; p < 0.001), high income of more than 1000 JDs (1400 USD) (OR = 3.23; 95% CI = 2.21-4.71; p < 0.001), having an educational level of either high education (OR = 3.39; 95% CI = 2.07-5.55; p < 0.001) or bachelor’s degree (OR = 1.67; 95% CI = 1.25-2.24; p = 0.001), and being encouraged by the increasing number of infections and deaths caused by COVID-19 (OR = 1.97; 95% CI = 1.46-2.66; p < 0.001) were certain factors associated with more likelihood of taking the vaccine.

**Table 4:** Multivariant regression analysis of the factors affecting the willingness to get the COVID-19 vaccine

Variables		Regression analysis		
		OR	95% CI	P-value
Age		0.98	0.97–0.99	0.002
Gender	Male	2.02	1.54–2.64	<0.001
	Female	0.50	0.38–0.65	<0.001
Income	Less than 500 JDs (700 USD)	0.31	0.21–0.45	<0.001
	500–1000 JDs (700-1400 USD)	0.62	0.40–0.95	0.027
	More than 1000 JDs (1400 USD)	3.23	2.21–4.71	<0.001
Residency	North territory	1.52	0.74–3.13	0.26
	Middle territory	1.59	0.86–2.95	0.14
	South territory	0.66	0.32–1.36	0.26
Educational level	High educational level	3.39	2.07–5.55	<0.001
	Bachelor	1.67	1.25–2.24	0.001
	Diploma or less	0.30	0.18–0.48	<0.001
Did not get COVID-19 infection		1.25	0.91–1.73	0.168
Having a member of family/friends infected with COVID-19		1.27	0.86–1.86	0.23
Being encouraged by reading scientific articles		1.20	0.92–1.57	0.177
Being encouraged by knowing the mechanism of action of the vaccine		0.65	0.50–0.84	0.002
Being encouraged by medical advice		1.06	0.79–1.44	0.69
Being encouraged by the high number of infections and deaths		1.97	1.46–2.66	<0.001
Being encouraged by knowing the side effects of the vaccine		0.50	0.34–0.71	<0.001
Being encouraged by the increasing number of people vaccinated against COVID-19		0.59	0.43–0.81	0.001

## Discussion

Immunization is a global health issue that saves millions of lives every year. Vaccines are crucial in preventing and controlling infectious disease outbreaks; COVID-19 vaccines are one of the best means to restrict the effects of the pandemic [19]. The demand for COVID-19 vaccines in Jordan is still below the desired level, mainly due to people's fear of side effects that may be caused by the vaccine [20]. Therefore, we think that this might be related to the humble educational level of most of our Jordanian population.

The educational level played a vital role in people's reluctance to COVID-19 vaccines [21], being one of the most important factors influencing people's decisions to refuse to take any vaccines due to their doubts about its ability to bring life back to normal. In general, people with high education showed more intention to take the vaccine. Our study and many others [22-24] confirmed this, which found that people with higher educational levels were more willing to take the vaccine. Thus, more efforts were needed to improve confidence regarding vaccination in lower education adults [21].

Our study showed that people with higher educational levels believed more in the vaccine's ability in routine life restoration. They were willing to pay for the vaccine even though it is not free compared to those with a bachelor's or less degree who were not. A previous study showed that the main reasons for which people are refusing to pay for the vaccine were the government should pay for it; the vaccine was unnecessary because the people do not have enough money, and they think that those who caused COVID-19 must pay for it [24].

Regarding the trusted vaccines, our study showed that people with high education and diploma or less would prefer to take Pfizer/BioNTech vaccine if they had the right to choose. In contrast, people with bachelor’s did not know which vaccine to take. This corresponds with Aloweidi et al.'s study, which found that non-medical personnel did not know which vaccine to choose between the available vaccines compared to the medical personnel [12].

Many variables showed significant results. Based on our findings, older people desired to take the vaccine more than younger ones. This was agreed by a global survey for potential acceptance of the COVID-19 vaccine in June 2020, which showed that people above 25 years were more likely to accept the vaccine than those in the age range of 18-24 years [25]. In contrast, concerning gender, our study showed that males were more willing to take the vaccine; this was confirmed by many studies [26,27] but was not consistent with the results of that global survey which showed men were slightly less likely to take it [25]. Regarding income, people with higher income were more likely to take the COVID-19 vaccine; however, people with lower-income households in the United Kingdom were more likely to reject the COVID-19 vaccine [28].

During the data collection period of this study, the number of daily new cases in Jordan rose from 730 patients on January 22, 2021, to 4594 cases on February 28, 2021. It continued to rise until it peaked at 9535 new cases on March 17, 2021 [29]. Interestingly, our results showed that people who had not been infected with the COVID-19 virus were more willing to get the vaccine, especially those who had a close person who previously got infected or dead due to COVID-19. On the contrary, Lazarus et al.'s study showed that the people who reported COVID-19 sickness in themselves or family members were less likely to respond positively to the vaccine question than other respondents [25].

Regarding the encouraging factors to get vaccinated, we found that people were more likely to take the vaccine if the number of vaccinated people got increased, which agreed with other studies that reported 82.3% of the participants would only take the vaccine if it were taken by many in public [30]. Moreover, knowing the vaccine's side effects helped encourage them to take it, which was illustrated in different studies and showed that one of their concerns in taking the vaccine was its side effects [27].

Information resources about COVID-19 vaccines are many, including scientific articles, internet pages, friends, and traditional media. Although a similar study in the Middle East found that social media had a different effect on the behavior of the population toward the virus or the vaccine [15], our study showed that people who took their vaccine knowledge from scientific evidence were the most willing to take the vaccine. It was reported that reading a scientific article about the available vaccines resulted in a significant increase in the willingness to take the vaccine [12].

The main limitation of this study was raised from the lack of previous studies made in this research field. Inequality in the distribution of age, gender, and occupation of the included sample may also cause statistical bias. Moreover, the data collection method focused mainly on the online survey; thus, many people were not accessible. The survey may be filled more than once by the same person so that the responses would be duplicated.

## Conclusions

The world has to improve people's confidence and willingness to take the vaccine, focusing on those with lower educational levels. Social media should be more attentive to the information provided for the public regarding medical issues in general and vaccines particularly, thereby changing the attitude and behavior toward COVID-19 and its vaccine by supplying people with the proper knowledge regarding the vaccine's mechanism of action, side effects, and efficacy.
